# Evaluation of the reach and utilization of the American College of Lifestyle Medicine’s Culinary Medicine Curriculum

**DOI:** 10.3389/fnut.2024.1338620

**Published:** 2024-03-19

**Authors:** Kara Livingston Staffier, Shannon Holmes, Micaela Cook Karlsen, Alexandra Kees, Paulina Shetty, Michelle E. Hauser

**Affiliations:** ^1^American College of Lifestyle Medicine, Chesterfield, MO, United States; ^2^Division of General Surgery, Department of Surgery, Stanford University School of Medicine, Stanford, CA, United States; ^3^Division of Primary Care and Population Health, Department of Medicine, Stanford University, Stanford, CA, United States; ^4^Internal Medicine-Obesity Medicine, Veterans Affairs Palo Alto Health Care System, Palo Alto, CA, United States

**Keywords:** culinary medicine, food as medicine, physician training, practitioner training, lifestyle medicine

## Abstract

**Introduction:**

Despite the growing interest in “food as medicine,” healthcare professionals have very limited exposure to nutrition as part of their training. Culinary medicine (CM), an evidence-based field integrating nutrition education with culinary knowledge and skills, offers one approach to fill this training gap. The American College of Lifestyle Medicine published a complimentary Culinary Medicine Curriculum (CMC) in 2019, and the objective of this study is to evaluate its reach and utilization, as well as to collect feedback from users.

**Methods:**

Individuals who downloaded the CMC prior to March 1, 2022 (*N* = 6,162) were emailed an invitation to participate in an online, cross-sectional survey. The survey included both multiple choice and free-text questions about whether CM sessions were conducted, if and how the CMC was used, if and how it was modified for use, and additional requested resources. Free-text responses were inductively coded, and quantitative data was summarized using descriptive statistics.

**Results:**

A total of 522 respondents provided consent, indicated that they had downloaded the curriculum, and completed the survey. Of the 522, 366 (70%) reported that they had not led or created any CM sessions. The top-reported reason for not leading a session was lack of time (29%). The remaining respondents who did create a CM session did so across various settings, including academic, clinical, coaching, and other settings, and a variety of professionals delivered the CMC sessions, including physicians (50%), registered dietitian nutritionists (30%), and chefs (25%). The majority of respondents (81%) modified the CMC in some way, with many using the curriculum for guidance or ideas only. Patient education materials (66%) and cooking technique instruction videos (59%) were among top requested resources.

**Discussion:**

The CMC is a versatile resource that can be successfully adapted for use across various settings and by various types of health professionals and practitioners. Future research should investigate whether training in CM results in improved health outcomes for patients/clients. The curriculum will continue to grow to address the needs of users by expanding to include more digital content such as curriculum videos and cooking technique videos.

## Introduction

Diet has been identified as the single most important risk factor for morbidity and mortality in the United States, yet most healthcare professionals spend relatively few hours learning about nutrition during their training (1, 2). Historically, nutrition education has been limited to fewer than 20 hours in the preclinical years of undergraduate medical education, focused on nutrients rather than food, and largely separated from the clinical experience (1, 2). Medical students report not feeling equipped to provide adequate nutrition care to patients, despite their acknowledgment that nutrition is a useful and necessary part of patient care (3).

To fill this void in nutrition training, the American College of Lifestyle Medicine (ACLM) made available the Culinary Medicine Curriculum (CMC) (4), in English, based on the Stanford University course developed by Dr. Michelle Hauser, to healthcare professionals via complimentary download in December 2019 (5, 6). Between December 2019 and March 2022, over 6,000 individuals from across the globe had downloaded the CMC. As of March 2024, the number of unique downloads had grown to over 10,000. The CMC was published for medical schools, health professional training programs, and residency programs with flexibility to be tailored to a variety of settings and audiences. The curriculum includes an instructor’s guide, recipes, shopping guides, and equipment lists for creating pop-up teaching kitchens. The beginning of the curriculum includes a section on nutrition not typically covered in medical and health professional schools that is critical for patient education and dietary behavior change.

Culinary medicine (CM) is an evidence-based field that integrates nutrition education with culinary knowledge and skills. The goal is to assist individuals in maintaining health and preventing and treating food-related diseases by choosing high-quality, healthy food in conjunction with appropriate medical care (6, 7). Foundational CM knowledge is characterized by understanding what constitutes a healthy diet and how to find, obtain, and prepare nutritious and delicious food to support improved health outcomes (6).

The recent growth of “food as medicine” (FAM), also called “food is medicine” (FIM), in research, public health, and clinical care has expanded the practitioner’s awareness of these concepts. While historically, FAM primarily referred to interventions providing food to patients, such as produce prescription programs and medically tailored meals (MTM), these types of programs may have limited sustainability without supporting education on food preparation (8). The field of lifestyle medicine (LM) focuses on treatment that employs behavior change education to help patients working to improve their health habits, and CM is a natural fit for this approach (9). FAM, as practiced by LM providers, is a key element of LM in the nutrition domain, and can serve to bridge LM and CM (10).

Programs at all levels of medical training are introducing CM educational opportunities to fill the void of practical nutrition skills and to better prepare health professionals to support patients in sustained, healthy dietary changes (2). The first culinary medicine course in the U.S. was Dr. John La Puma’s at the State University of New York-Upstate campus in 2003. The first CM continuing medical education conference (Healthy Kitchens, Healthy Lives [HKHL]) was held in 2007, co-sponsored by Harvard Medical School and the Culinary Institute of America in St. Helena, California. In 2012, the first permanent teaching kitchen was established in a medical school at the Goldring Center for Culinary Medicine (GCCM) at Tulane University School of Medicine, led by physician-chef Timothy Harlan (11). This program has grown dramatically and since then, over 30 U.S. medical schools throughout the country have implemented programs (12–14). In addition, other CM programs have been implemented in health systems (15) or community or patient care settings (16–19).

The CMC continues to be offered and has been utilized in the form of a teaching kitchen elective course for medical and physician assistant students at Stanford University School of Medicine and showed significant improvements in attitudes, knowledge, and behaviors around healthy cooking and meal planning (4). Other studies on CM educational programs for health care professionals in training have demonstrated significant and positive impacts on medical students’ attitudes, knowledge, and competencies with practical, hands-on culinary skills and nutrition knowledge (12, 20–27).

The objective of this study was to evaluate the use of the ACLM’s CMC, as follows: (1) characterize respondents who have utilized the CMC, (2) understand how they have used the CMC, (3) identify modifications that have been made to the curriculum during implementation in academic and clinical settings, and (4) gather information on the challenges and needs of curriculum implementers for the purpose of informing further resource development efforts.

## Methods

Individuals who downloaded the CMC from its inception in December 2019 to March 1, 2022 were emailed an invitation to participate in a cross-sectional survey (*n* = 6,162). The survey was administered in English, using QuestionPro, a secure, online survey platform and open from March 2–April 22, 2022. This study was administered by ACLM and reviewed by the University of New England Institutional Review Board. All respondents provided informed consent, and all research team members completed training in human subjects research by the Collaborative Institutional Training Initiative (CITI).

The survey was developed by members of the research team (SH, PS, MEH) based on interest in learning about the CMC for the main purpose of quality improvement. The survey was not pilot-tested or validated, and no scoring or classification procedure was applied. The first three questions were used to screen that respondents were at least 18 years of age, understood the study as described in the consent, and agreed to participate. Individuals answering “yes” to these questions were eligible to participate. The remainder of the survey consisted of a maximum of 18 questions, using programmed logic to display only relevant questions. The survey included questions on clinical degree, certification and/or training when the curriculum was downloaded, and whether respondents had created any CM sessions. For those who created one or more CM session(s), follow-up questions were asked on the primary way in which the CMC was used, approximate number of students/patients in each session, number of sessions conducted, primary setting in which the curriculum was used, and who led and/or taught the sessions (i.e., MD, RDN, health coach, etc). Respondents were also asked for details about if and how they used the CMC in creating their sessions, including if and how they modified the curriculum and whether they would have liked additional resources or materials available with the curriculum. Both multiple-choice and free-text questions were used. Respondents who reported not using the CMC were asked to explain why. Supplementary Table 1 contains a summary of the survey questions. The survey was set to only allow it to be completed one time, which is achieved through cookies saved on the browser. The data was additionally reviewed to ensure that two responses with the same email address were not recorded. If so, only the most complete response was retained. Survey responses were confidential, with datasets being stored on a secured server with access restricted to only study team members. In addition, identifying information (name, email) was removed prior to analysis.

Quantitative data were summarized using descriptive statistics. Missing data were not imputed. As this was a descriptive analysis aimed at capturing feedback from individuals who downloaded the curriculum for the purpose of quality improvement and programmatic development, sample size and power calculations were not performed. Two researchers independently inductively coded free-text data to identify emergent categories of responses, and discrepant coding was resolved through discussion with a third member of the research team to modify codes as needed and achieve consensus. Categories of free-text responses were descriptively summarized. Analyses were conducted using SAS version 9.4.

## Results

**Figure 1:**
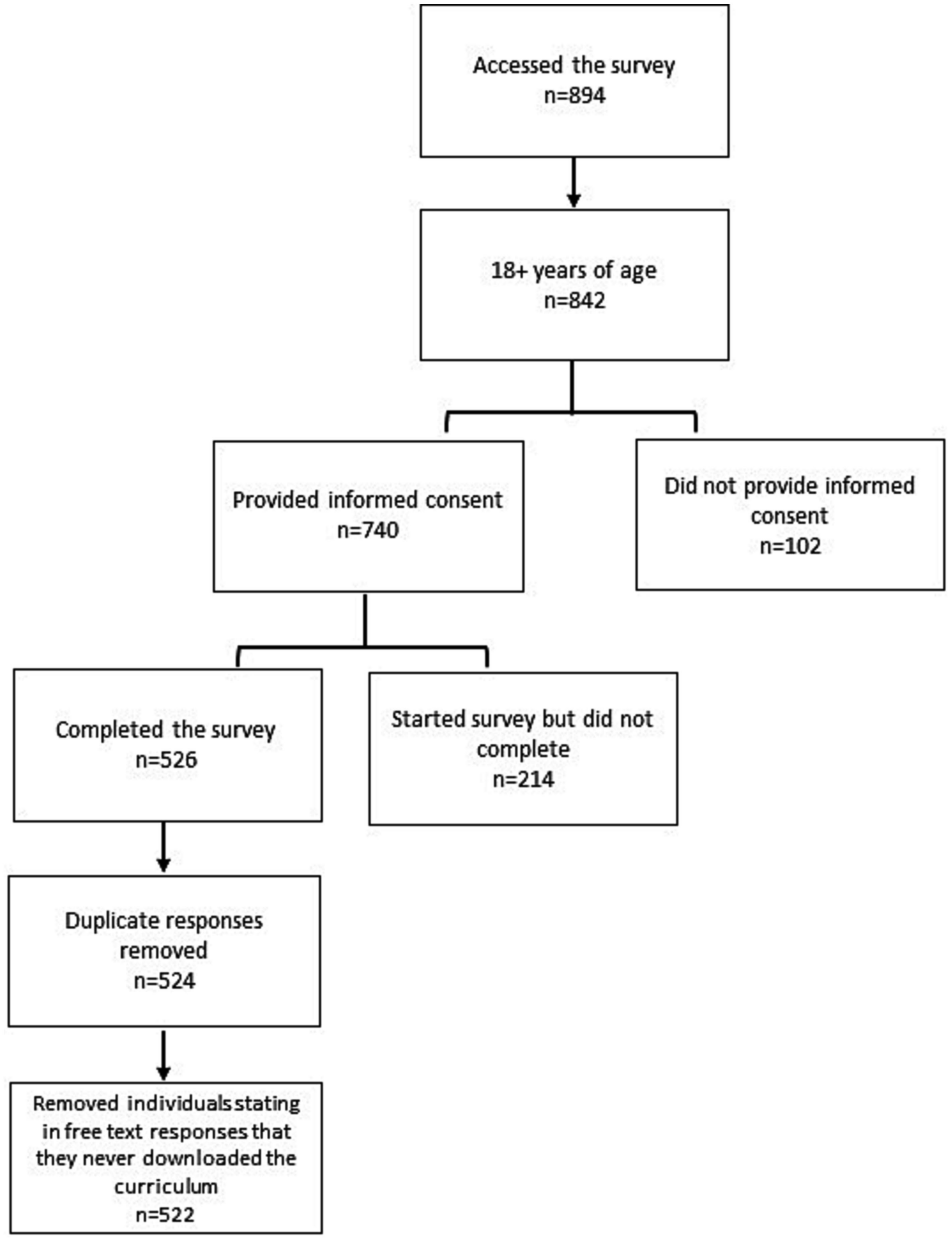
Participant enrollment and completion.

The online survey was accessed 894 times, and 83% (*n* = 740) of respondents were at least 18 years old and provided informed consent to participate. Of those providing consent, 71% (*n* = 522) completed the survey after removing duplicate responses (*n* = 2) and individuals (*n* = 2) stating in free-text responses that they had never downloaded the curriculum (Figure 1).

The majority of respondents (47%) reported having a medical degree (MD or DO); 13% reported a nursing credential, 48% of which were advanced practice registered nurses; 30% percent reported credentials in another health professional field (non-MD/DO, non-nursing); and 29% reported a non-clinical credential. Non-clinical credentials reported included chefs, public health degrees, academic doctoral degrees, and degrees in business and education. Twenty-three percent of respondents were certified in LM (DipABLM, DipIBLM, or DipACLM). Eighty-two percent (*n* = 430) were from the United States with respondents from 38 other countries represented.

Twenty-three percent had downloaded the curriculum within the past 3 months at the time they were surveyed; however, 35% had downloaded the curriculum more than 1 year prior. Of the 522 respondents in the final sample, a total of 366 (70%) reported that they had not created any CM sessions at their institution or practice. The top reasons reported included lack of time (29%), the program at their organization for which the CMC could be used was still in development (13%), the Covid-19 pandemic (12%), no opportunity (12%), and lack of funding or resources (10%). Lack of interest by patients or clients was reported by only 3% of respondents (Table 1). The remaining 149 (29%) respondents reported that they created a CM session.

**Table 1:** Participant characteristics and creation of culinary medicine sessions.

Licensure/credentials (*n* = 522)^a^	
Physician (MD/DO)	247 (47.3)
Nursing^b^	65 (12.5)
Other patient care field (excludes MD/DO and nursing)	158 (30.3)
Non-clinical	152 (29.1)
Social work	8 (1.5)
Certified in lifestyle medicine (*n* = 522)	120 (23.0)
Country (% from United States) (*n* = 522)^c^	430 (82.4)
How many months ago downloaded the CMC (*n* = 522)^d^	
0–3	119 (22.8)
3–6	85 (16.3)
6–12	102 (19.5)
12–24	121 (23.2)
>24	63 (12.1)
Not reported	32 (6.1)
Created a CM session at their institution or in their practice (*n* = 522)^e^	
Yes	149 (28.5%)
No	366 (70.1%)
Why have you not led or created any culinary medicine sessions? (n = 362)^f^	
Lack of time	104 (28.7%)
Program is in progress but not completed/in development	46 (12.7%)
Covid-19 pandemic	45 (12.4%)
No opportunity	44 (12.2%)
Lack of funding/resources	37 (10.2%)
Do not yet feel qualified/prepared to lead a session	28 (7.7%)
Lack of institutional support/organizational issues	28 (7.7%)
Did not answer question (not codable)	28 (7.7%)
Not relevant/unsure of relevancy to current institution/clinic/course/job	18 (5.0%)
Lack of interest by patients or clients	11 (3.0%)
Using a different curriculum/curriculum being developed by someone else	8 (2.2%)
Using curriculum for other use: clinical purposes, personal use, etc.	7 (1.9%)

^a^*n* = 117 (22.4%) respondents overall reported multiple licensures/credentials, and *n* = 51 (20.6%) of physicians reported multiple licensures/credentials; ^b^*n* = 31 (47.7%) were advanced practice registered nurses; ^c^Other top countries reported by respondents were as follows *n* (%): Canada 14 (2.7%), Philippines 9 (1.7%), United Kingdom 8 (1.5%), and Brazil 6 (1.2%); ^d^At the time of data collection: 3/2/2022 to 4/22/2022; ^e^*n* = 7 missing/prefer not to answer; ^f^Responses coded into multiple categories where applicable.

Among the 149 respondents that created a session, 113 reported on number of CM sessions run and number of participants in a session. These numbers varied widely among respondents, ranging from 0 to 186 total sessions run [median (IQR) = 4.0 (7.0)] and 0–800 participants in each session [median (IQR) = 10.0 (16.5)]. When asked to identify the primary way in which the curriculum was used, 48% reported using it as inspiration or support for materials that the respondent created, while 14% used the CMC and substantially modified materials. Approximately 3% used all sessions with few or no modifications, and 23% did not use the CMC when leading or creating a session. These respondents reporting that they did not use the CMC (*n* = 34) identified the following reasons for non-use: used a different curriculum (50%) or did not yet review the curriculum as a resource (24%), the CMC was not relevant (9%), or the respondent did not have decision-making authority over the curriculum (6%).

One-hundred-thirteen respondents who created a session and used the CMC in some capacity reported further detail on the curriculum setting. Specifically, 39 (35%) used the curriculum in an academic setting, 31 (27%) in a clinician-patient care setting, and 26 (23%) in a client coaching setting (Table 2). Among 39 academic setting users, the curriculum was most frequently implemented in medical school (56%) and graduate medical education such as residency and fellowship (23%) settings. The academic learning environment was 39% hybrid (combined in-person and online), 39% online (36% synchronous, 3% asynchronous), and 23% in-person sessions. Among 31 clinical setting users, the curriculum was most frequently implemented in LM (42%) practices, followed by family medicine (16%), internal medicine (13%), and preventive medicine practices (10%). Another 19% of respondents reported using the curriculum in a combination of ‘other’ practice types including pediatrics, cardiology, and oncology. The clinical environment was 48% private practice, 45% health system setting, 16% academic practice, and 7% other settings, including one federally qualified health center (3%) (Table 2).

**Table 2:** Use of the Culinary Medicine Curriculum among respondents who created a session.

What was the primary way in which you used the Culinary Medicine Curriculum in your sessions? (*n* = 149)^a^	
I used the CMC as inspiration or for support of materials that I created	72 (48.3)
I did not use the CMC in any way when leading or creating my CM session(s)	34 (22.8)
I used the CMC but modified the materials substantially for my purposes	21 (14.1)
I used 1 or more sessions from the CMC with few or no modifications	16 (10.7)
I used all nine sessions from the CMC with few or no modifications	4 (2.7)
If did not use, why did you not use the CMC? (*n* = 34)	
Used a different curriculum that was already designed	17 (50.0)
Haven’t yet reviewed the curriculum or did not think to review/use it	8 (23.5)
Other^b^	4 (11.8)
CMC was not relevant to the course	3 (8.8)
Curriculum content was not my decision	2 (5.9)
In what primary setting did you use the curriculum (*n* = 113)^c^	
Academic: teaching students and trainees	39 (34.5)
Primary setting curriculum used in (*n* = 39)^d^	
Medical school	22 (56.4)
Graduate medical education (residency, fellowship)	9 (23.1)
Continuing education/continuing medical education	4 (10.3)
Pre-professional (bachelor’s, associate’s, trade program)	4 (10.3)
Culinary	3 (7.7)
Dietetics	2 (5.1)
Master’s program	2 (5.1)
Nurse practitioner or physician associate/assistant	3 (7.7)
Nursing (LVN, RN)	1 (2.6)
Psychology or PhD	1 (2.6)
Type of learning environment (*n* = 39)	
Hybrid (part in-person and online)	15 (38.5)
Online, scheduled sessions	14 (35.9)
In-person sessions	9 (23.1)
Online, on-demand sessions	1 (2.6)
Clinical: patient care	31 (27.4)
Type of practice (*n* = 31)	
Lifestyle medicine	13 (41.9)
Other^e^	6 (19.4)
Family medicine	5 (16.1)
Internal medicine	4 (12.9)
Preventive medicine	3 (9.7)
Practice environment (*n* = 31)^d^	
Private practice	15 (48.4)
Health system	14 (45.2)
Academic practice	5 (16.1)
Other^f^	2 (6.5)
Clients/coaching	26 (23.0)
Other	16 (14.2)
Prefer not to answer	1 (0.9)
Who leads and/or teaches the culinary medicine sessions (*n* = 113)^c,d^	
Physician (MD/DO)	57 (50.4)
Registered dietitian nutritionist (RDN)	34 (30.1)
Chef	28 (24.8)
Health coach	22 (19.5)
Health educator	12 (10.6)
Other	12 (10.6)
Advanced practice provider (NP, PA)	9 (8.0)
Nurse (RN, LVN)	8 (7.1)
What additional materials or resources would be helpful to have as part of the curriculum or to support the curriculum? (*n* = 113)^c,d^	
Patient education materials	75 (66.4)
Cooking technique instruction videos	67 (59.3)
Nutrition education curriculum	61 (54.0)
Online culinary medicine course for continuing education	50 (44.2)
In-person culinary facilitator training	30 (26.6)
More detailed case studies	28 (24.8)
Other	12 (10.6)

^a^*n* = 2 selected prefer not to answer; ^b^includes not enough interest, not enough training, missing response; ^c^*n* = 2 who selected “prefer not to answer” regarding the primary way that they used the curriculum and *n* = 34 indicating “I did not use the CMC in any way when leading or creating my CM session(s)” excluded from denominator; ^d^Responses coded into multiple categories where applicable; ^e^Pediatrics, cardiology, oncology, other unspecified; ^f^Federally qualified health center or other (unspecified).

Sessions were most frequently led by physicians (MD/DO, 50%), followed by registered dietitian nutritionists (RDNs, 30%), chefs (25%), and health coaches/health educators (30%). The majority of respondents who led a session reported that additional materials or resources would be helpful to support the curriculum. This included patient education materials (66%), including videos, recipes, handouts or infographics, followed by cooking technique instruction videos (59%), and additional nutrition education curriculum (54%).

Eighty-one percent of respondents (*n* = 113) who used the CMC in some way modified the curriculum. The following modifications were reported: reducing the number of sessions (37%), adding content to the curriculum (34%), removing content from the curriculum (22%), and creating more than 9 sessions either by dividing course material into smaller sessions and/or adding additional content of their own (7%) (Table 3). Respondents were additionally asked to explain their curriculum modifications in greater detail using free text, and answers were inductively coded. Forty-three percent reported using the CMC for guidance/ideas only, 25% changed the CMC to fit the needs of a specific community, culture, or population, 9% shortened or reduced sessions or materials, 9% included additional information or materials, and 7% adapted the CMC to a virtual format.

**Table 3:** Modification of the Culinary Medicine Curriculum.

Did you modify the CMC?^a^ (*n* = 113)	
Yes	91 (80.5)
How did you modify the curriculum? (*n* = 91)	
Number of sessions was altered from 9 to fewer	34 (37.4)
Content was added to the curriculum	31 (34.1)
Content was removed from the curriculum	20 (22.0)
Number of sessions was altered from 9 to greater	6 (6.6)
Please explain your modifications in detail (*n* = 91)^c^	
Used the curriculum for guidance or ideas only	39 (42.9)
Changed curriculum to fit community, cultural, or local needs	23 (25.3)
Other^b^	10 (11.0)
Changed format- shortened	8 (8.8)
Included additional information/materials	8 (8.8)
Changed format to virtual	6 (6.6)
No	15 (13.3)
Prefer not to answer	7 (6.2)

^a^*n* = 2 who selected “prefer not to answer” regarding the primary way that they used the curriculum and *n* = 34 indicating “I did not use the CMC in any way when leading or creating my CM session(s)” excluded from denominator; ^b^Other (unspecified), not codable; ^c^Responses coded into multiple categories where applicable.

## Discussion

The CMC has emerged as a valuable and flexible resource in the fields of CM and LM. This evidence-based curriculum has seen a substantial increase in number of downloads since its launch reflecting the importance of CM in bridging the knowledge gap for nutrition and LM education among healthcare professionals, students, and non-practitioners alike. A growing body of research indicates that CM programs increase practitioner confidence in nutrition knowledge and skills (13, 24, 28, 29), as well as ability to counsel patients (30) and help patients overcome barriers to healthy eating (13). In addition, CM programs can improve cardiometabolic outcomes in patients (31), as well as improve personal culinary skills (32). The importance of practitioners-in-training improving their personal culinary skills is emphasized in research that suggests medical students and residents with personal experiences following a plant-based diet expressed greater willingness to recommend it to patients (33). The current study is unique, however, in that no other study to our knowledge details how a CM program is utilized and modified in a real-world setting.

The results of this survey suggest that a major strength of the CMC is its adaptability and flexibility to be used across diverse settings and be led by different individuals, making it a versatile tool to integrate FAM into practice for educators, healthcare providers, and community leaders. Its adaptability enables customization to meet the specific needs and goals of users, further enhancing its utility. Respondents used the curriculum across academic, clinical care, and client/coaching settings and in a variety of forms, specifically online, in-person, and hybrid settings. This flexibility was important given that the curriculum was made available in December 2019, and many of the respondents surveyed were accessing the curriculum during the COVID-19 pandemic. Other published research has shown positive outcomes across both in-person and virtual CM teaching platforms (13, 17, 34). Within clinical settings, use was not just restricted to LM practice (42%) but extended into other medical specialties such as family medicine (16%), internal medicine (13%), and preventive medicine (10%). The integration of lifestyle approaches across these clinical fields through the use of CM is encouraging and consistent with other published literature highlighting the utility of CM across different fields such as women’s health (34), pediatrics (19), oncology (16), and in the care of patients with diabetes (17, 31).

Approximately one-quarter of respondents who had created a session adapted the curriculum to fit the unique needs of their community, such as modifying the curriculum to a specific culture, population, or dietary pattern. Tailoring curricula to specific cultural needs is of critical importance, as racial and ethnic minorities in the United States suffer disproportionately from diet-related chronic disease (35). In addition, chronic disease burden is higher and multimorbidity, defined as having two or more coexisting chronic conditions, starts at an earlier age in Hispanic/Latino and African American populations compared to their white counterparts (36). Incorporating culture into nutritional counseling may incite greater adherence to dietary changes (37) and, consequently, promote better health. For example, a recent study showed that a cookbook tailored to a Filipino-American population may potentially motivate individuals to make healthier dietary choices (38).

LM practitioners utilize a team approach, incorporating non-physicians such as nurses, registered dietitians, and wellness coaches (39, 40). The importance of a team approach and the potential for expanded patient reach is further highlighted by the broad array of expertise among those leading/teaching the sessions. While half of those reported to be leading and/or teaching sessions were physicians (50%), registered dietitian nutritionists (30%), health coaches/health educators (30%), and chefs (25%) were frequently reported. Similarly, a 2021 review of nutrition education interventions noted the importance of interprofessional learning (41). This broad reach among health professionals represents the CMC as useful for various professional and clinical backgrounds.

Lack of knowledge and time have been reported as reasons for not providing nutrition education to patients (42). This curriculum is a free, publicly available resource that was created in response to interest in creating CM sessions among individuals who do not have the training and skills to develop their own curriculum from scratch. The CMC is intended to promote greater confidence in creating sessions and, thus, empower interested individuals to lead CM sessions addressing barriers of skills and time. Despite this, the survey results indicate that time constraints remain a significant barrier to implementation, with time overwhelmingly identified as the top reason for not leading a session. This highlights the need for continued support and resources to assist healthcare professionals and educators in finding not just time, but implementing strategies for successful reimbursement allowing for compensated time that could be devoted to fully integrating CM into their practice and setting.

While other reasons for not leading a session varied widely, several reasons cited were related to the infrastructure, specifically lack of funding/resources and lack of institutional support/ organizational issues. As Mauriello and Artz discuss, CM can only improve health and reduce healthcare costs when it is fully integrated into the healthcare model. Medical student training alone is not enough (43). There is a need for more documented examples of successful integration of CM programs into a variety of practice settings. Such implementation models could support the documentation of best practices for allocating time and resources for CM curricula in general.

Not surprisingly, the additional materials and resources that were identified as being potentially beneficial to have as part of the curriculum varied, with patient resources and additional information emerging as top themes. Examples of additional requested patient resources included videos (cooking techniques, nutrition), recipes, handouts and infographics. Requests for additional information included clinical cases/case studies and evidence-based nutrition education. To a smaller extent, additional education was requested including on-site facilitator training, and cooking technique instruction videos. The CMC is in the process of being modified to meet the expanding needs of those currently utilizing it as expressed in this survey. Course videos including cooking technique videos and recipe instruction videos for all recipes presented in the curriculum have been filmed. Eight faculty interviews covering the topics of nutrition education and dietary behavior change counseling have also been filmed, as well as eight or more patient case examples with expert faculty counseling and recommendations on dietary behavior change incorporated. All videos are currently in the post-production phase. Finally, even with these additions, it is likely that modification of nutrition education resources like the CMC will still be needed to tailor the curriculum to the patient population. This is a strength of providing a free resource with the potential to be altered for the situation.

A limitation of this analysis is that the recruited sample only represents a proportion of the population that accessed ACLM’s CMC; the sample that completed the survey is a small proportion of that group and an even smaller number reported creating a CM session. In addition, the CMC assessed in this study highlights a whole-food, plant-predominant diet, as recommended by ACLM. Thus, results may not be generalizable to the overall population accessing this specific curriculum or utilizing other CM programs. However, since ACLM is a medical professional organization that uniquely serves not only physicians but all healthcare professionals, the CMC is available to a diverse audience including nurses, dietitians, health coaches, and other health professionals, all of which were reflected in the survey respondent base. Additionally, the environments available to utilize the curriculum may have been affected by the COVID-19 pandemic. While the pandemic may have created time barriers and disrupted the implementation of certain programs, it may have also facilitated the transition to successful virtual programs. However, the survey’s design and scope do not allow for a comprehensive assessment of these specific effects. Finally, nonresponse bias at the survey level cannot be addressed within the scope of the approved study protocol, which allowed for use of data collected in the survey, as opposed to the other, limited demographic data originally collected upon download. We do have limited data on the credentials/training of survey completers v non-completers (Supplementary Table 2); however, the majority of non-completers did not provide data, making it difficult to assess differences between the groups. Despite these limitations, it is crucial to recognize the pioneering nature of this study, assessing real-world use of the first and only open-source CM curriculum made widely available.

Significant room for improvement exists for better equipping practitioners to address diet and lifestyle with their patients. The CMC has proven to be an asset in the promotion of CM, FAM, and LM. Its broad reach, adaptability, and flexibility position it as a pivotal tool in the quest to improve nutrition education and, subsequently, the overall health and well-being of patients. Future research should assess practitioner confidence and knowledge after completing the CMC. Additionally, since the survey did not assess SMAs or reimbursement approaches, the use of shared medical appointments (SMAs) should be explored as a strategy for creating the compensated time required to deliver the curriculum. Additional research is needed to determine the ability of this particular curriculum to impact health behaviors and outcomes, specifically whether CM training results in improved health outcomes for patients/clients. Continued efforts to address the identified barriers to running CM sessions will be essential in realizing the full potential of this valuable resource.

## Data availability statement

The datasets presented in this article are not readily available because the IRB has not approved the release and/or use of this data for individuals not on the research team. Requests to access the datasets should be directed to MK, mkarlsen@lifestylemedicine.org.

## Ethics statement

The studies involving humans were approved by University of New England Institutional Review Board. The studies were conducted in accordance with the local legislation and institutional requirements. The participants provided their written informed consent to participate in this study.

## Author contributions

KS: Data curation, Formal analysis, Writing – original draft. SH: Writing – original draft. MK: Conceptualization, Supervision, Writing – review & editing. AK: Writing – review & editing. PS: Conceptualization, Supervision, Writing – review & editing. MH: Conceptualization, Supervision, Writing – review & editing.
