# Supplementary material for: Evaluation of the reach and utilization of the American College of Lifestyle Medicine’s Culinary Medicine Curriculum
Source: Front Nutr. 2024 Mar 19;11:1338620. doi: 10.3389/fnut.2024.1338620 (PMC10985187; doi:10.3389/fnut.2024.1338620)
Supplement: Supplementary file 1 [file Table_1.docx]

**Supplemental Table 1: Summary of Survey Questions**

| Please indicate your credentials/licensure | Multiple Choice (select all) |
| --- | --- |
| How long ago did you download the curriculum | Multiple Choice (single select) |
| Have you led or created any CM sessions at your institution or in your practice?    If no: why have you not led or created any sessions? | Multiple Choice (single select)        Free text response |
| What was the primary way in which you used the CMC in your sessions?    If selected CMC was not used: why did you not use the CMC when leading or creating your sessions? | Multiple Choice (single select)      Free text response |
| Approximately how many students or patients participated in each session of the course that you ran? | Fill-in (numeric) |
| How many sessions of the course have you run in total? | Fill-in (numeric) |
| In what primary setting did you use the curriculum?    If academic setting was selected:  Type of program  Learning environment    If clinical setting was selected:  Type of practice  Practice environment | Multiple Choice (single select)      Multiple Choice (select all)  Multiple Choice (single select)      Multiple Choice (single select)  Multiple Choice (select all) |
| Who leads and/or teaches the CM session(s)? | Multiple Choice (select all) |
| Did you modify the curriculum?    If yes: How did you modify the curriculum?    If yes: Please explain your curriculum modifications in detail | Multiple Choice (single select)    Multiple Choice (single select)    Free text response |
| What additional materials or resources would be helpful to have as part of the curriculum or to support the curriculum? | Multiple choice (select all) |

**Supplemental Table 2: Credentials/training^a^ of respondents who completed vs. did not**

**complete the survey**

|  | **Completed survey**  **(n=526)** | **Started but did not complete survey (n=214)** |
| --- | --- | --- |
| MD/DO | 247 (47.0%) | 17 (7.9%) |
| Other health professional (non-MD/DO; non-nursing) | 161 (30.6%) | 7 (3.3%) |
| Non-clinical/non-patient care | 153 (29.1%) | 7 (3.3%) |
| Social work | 8 (1.5%) | 0 (0.0%) |
| Nursing (NP, APRN, RN) | 67 (12.7%) | 0 (0.0%) |
| Certified in lifestyle medicine (DipABLM, DipIBLM, DipACLM) | 121 (23.0%) | 9 (4.2%) |
| Missing/no response | 0 (0.0%) | 185 (86.5%) |

^a^multiple selections allowed
